# EnhancerDB: a resource of transcriptional regulation in the context of enhancers

**DOI:** 10.1093/database/bay141

**Published:** 2019-01-24

**Authors:** Ran Kang, Yiming Zhang, Qingqing Huang, Junhua Meng, Ruofan Ding, Yunjian Chang, Lili Xiong, Zhiyun Guo

**Affiliations:** School of Life Sciences and Engineering, Southwest Jiaotong University, Chengdu City, Sichuan Province, P.R. China

## Abstract

Enhancers can act as *cis*-regulatory elements to control transcriptional regulation by recruiting DNA-binding transcription factors (TFs) in a tissue-specific manner. Recent studies show that enhancers regulate not only protein-coding genes but also microRNAs (miRNAs), and mutations within the TF binding sites (TFBSs) located on enhancers will cause a variety of diseases such as cancer. However, a comprehensive resource to integrate these regulation elements for revealing transcriptional regulations in the context of enhancers is not currently available. Here, we introduce EnhancerDB, a web-accessible database to provide a resource to browse and search regulatory relationships identified in this study, including 131 054 581 TF–enhancer, 17 059 enhancer–miRNAs, 318 993 enhancer–genes, 4 639 558 TF–miRNAs, 1 059 695 TF–genes, 11 439 394 enhancer–single-nucleotide polymorphisms (SNPs) and 23 334 genes associated with expression quantitative trait loci (eQTL) SNP and expression profile of TF/gene/miRNA across multiple human tissues/cell lines. We also developed a tool that further allows users to define tissue-specific enhancers by setting the threshold score of tissue specificity of enhancers. In addition, links to external resources are also available at EnhancerDB.

## Introduction

Enhancers are distal *cis*-regulatory DNA elements that positively regulate the transcription of target genes in a tissue-specific and spatiotemporal-specific manner (1). They often contain specific sequences acting as substrates for binding of tissue-specific transcription factors (TFs) and modulating expression of target genes (2). Recent studies have shown that TFs play key roles in regulation of enhancer activation, controlling enhancer function and even modulating chromatin accessibility in defined enhancer regions (3, 4). In some cases, mutations in the enhancer region can change the binding sites for TFs, resulting in the gain or loss of transcription regulation and participating in the occurrence of diseases such as cancer (5). Under the control of TFs, enhancers usually perform their transcriptional regulatory functions through targeting upstream and downstream target genes. In the past, target genes of enhancers were mainly considered as protein-coding genes. However, a recent study showed that enhancers can regulate the expression of adjacent microRNAs (miRNAs) and participate in the biological synthesis of miRNAs (6), suggesting that the enhancer-mediated regulatory network is much more complicated than it was known. Therefore, genome-wide identification and characterization of the regulation relationships between enhancers and the regulatory elements mentioned above across multiple tissues are necessary to reveal transcriptional regulations, developmental regulation and cell identity in enhancer contexts.

Based on genomic characteristics of enhancers, researchers have recently developed several enhancer databases such as VISTA enhancer (7), SEA (8), dbSUPER (9) and EnhancerAtlas (10) to help biologists to explore the enhancers. Although these databases do very well, they mainly focus on the identification and functional annotation of the enhancers using different approaches. However, it is also important to explore the regulation relationships between the upstream/downstream enhancer regulators and enhancers across multiple tissues to figure out the mechanisms of how enhancers perform their regulatory functions in the entire network. DENdb (11) is the first database concerned with enhancer regulatory relationships, including TF-enhancer and enhancer-gene relations. Conversely, the EnhancerDB we developed mainly focus on newly discovered regulation between enhancers and miRNAs and the regulatory relationship between TFs and enhancers/miRNAs. Recent research suggested that central roles of single-nucleotide polymorphisms (SNPs) located within enhancers or affect TF binding sites (TFBSs) located on enhancers in the regulation of diseases (12, 13). Therefore, EnhancerDB-integrated expression quantitative trait loci (eQTL) SNPs and SNPs that influence the binding of TFs, as well as the expression profiles of miRNAs and genes, all of which are not provided by DENdb. EnhancerDB contains data of 41 tissues/cell lines, much more than the 15 cell lines that DENdb provides. Moreover, with an increasing amount of data for histone markers such as DNase I hypersensitive sites (DHS), ChIP-seq, gene expression and eQTLs, there is an urgent need of building a comprehensive enhancer regulation database to integrate all these emerging omics data in various types of normal and cancer tissues/cell lines.

Previous studies revealed that active enhancers are always associated with DHSs, a hallmark of chromatin regions sensitive to the binding of TFs (14), and other histone modification features such as high levels of H3K27ac and H3K4me1 as well as low level of H3K4me3 (1). Fortunately, nowadays, a large number of omics data in tissues or cell lines are available including histone modification, DNase-seq, TF ChIP-seq, miRNA, gene expression, SNPs and eQTLs data. Since these data all have the characteristics of tissue-specific distribution, it is better to show the putative regulatory relationships by integrating data originating from the same cell line or tissue. For instance, to identify the regulation between TFs and enhancers in HepG2, one is supposed to integrate both the ChIP-seq data and histone modification data in HepG2, rather than the ChIP-seq data from HepG2 and histone modification data obtained from other cell lines. The latter approach tends to produce false-positive results because a large number of the identified regulatory relationships were not really involved in a specific tissue. In order to avoid similar problems, we tried our best to identify positive regulatory relationships involved in a certain tissue by using omics data only presented in the same tissue under the condition of limited data sets. In this study, we used general method for enhancer identification by combining tissue-specific histone modifications, namely H3K27ac, H3K4me1 and H3K4me3, with DHS data from ENCODE and GEO database, (15, 16). In addition, the regulatory element data for TFs, miRNAs, genes, expression profiles, SNPs and eQTLs were integrated to elucidate the regulation relationships between enhancers and these elements in multiple tissues/cell lines. Furthermore, the tissue-specific expressions of genes and miRNAs were also catalogued in database for understanding specific regulations in a certain tissue. Finally, we provided a user-friendly online platform named EnhancerDB for storing, analyzing and displaying the regulation relationships in the context of defined enhancers. We present EnhancerDB database that provides (i) identification of human enhancers in multiple normal or cancer tissues/cell lines; (ii) identification of the TF–enhancer, enhancer–miRNA, enhancer–gene, TF–microRNA and TF–gene interaction and related expression regulation across multiple tissues/cell lines; (iii) the potential impact of SNPs within the enhancer or affecting TFBSs located on an enhancer; and (iv) tools for users to screen customized confidence and specific/ubiquitous enhancers and links to external useful resources.

## Materials and methods

### Data sources

Histone modifications, DNase-seq data of 41 human tissues/cell lines, were downloaded from ENCODE and GEO databases (Supplementary data Table S1). The details of data sources of TFBS, gene, miRNA, SNP and eQTL were listed in Table 1. The expression values were normalized to Transcripts Per Kilobase (TPM) value, and genomic locus were converted to hg19. In order to screen highly conserved TFBSs from UCSC Txn factor track, only those with score higher than 800 were reserved. For TFs with no available ChIP-seq, the TFBSs were predicted using tfscan (17) and MOODs (18).

**Table 1:** The source and number of different types of data

	Data type	Source	Number of records	Version
TFBS	ChIP-seq	CistromeDB (21)	4 807 970	
		GTRD (22)	2 194 838	
		UCSC ENCODE uniform TFBS	990 356	
		UCSC Txn factor track	508 553	
	Predicted	JASPAR (23)	42 431 172	
		TRANSFAC (24)	262 793 871	
Gene	Expression value	GTEx portal (25)	244 088	
		HPA	68 308	
		ENCODE (26)	436 281	
	Annotation	GENCODE (27)		v19
miRNA	Expression value	FANTOM (28)	5784	
		ENCODE	2496	
		microRNA.org (29)	240	
	TSS	FANTOM (28)		
	Annotation	miRBase (30)		v20
SNP		dbSNP	11 381 519	Human b150 20170710 common
eQTL		GTEx protal	23 334	v7
Enhancer		FANTOM (28)	65 423	
		VISTA enhancer (7)	1835	

### Identification of enhancers

The enhancers were identified through combining DHS and histone markers based on the general method described previously, (15, 16). The enhancers were identified as the following criteria: (i) the H3K27ac, H3K4me1, H3K4me3 and DNase-seq signals were normalized using the following formula:

$$ \mathrm{Normalized}\ \mathrm{signal}=\frac{S\times {10}^9\kern0.5em }{\mathrm{Sum}\left(\mathrm{S}\right)}. $$

S represents the signal of each site on the reference genome. The signal peak region will be recentered if the peak was not located in the center of the signal peak region. (ii) We defined the 2 kb upstream and downstream from DHS center as candidate enhancer regions. (iii) The candidate enhancers will be reserved if the region of each enhancer exhibited high levels of H3K4me1 and H3K27ac and a low level of the H3K4me3 signals. (iv) The enhancers with genomic location overlapping with 5 kb upstream and 1 kb downstream from transcriptional start site (TSS) and the exon region of gene were discarded. (v) The position of ChIP-seq peak may not be very accurate due to ChIP-seq experimental processing and post-analysis errors. Therefore, we considered the identified enhancers from multiple tissues/cell lines as the same enhancer if they overlap at the genome positions and clustered them as the final identified enhancers. Moreover, because the accuracy of the identification of active enhancers depends on the high H3K27ac signal and high H3K4me1/H3K4me3 ratio (19), we designed the signal ratio to measure the identification accuracy of enhancers. The raw ratio of each enhancer was calculated based on the following formula:

$$ Rr=\frac{R1\times R2}{R3} $$

$$ Signal\ ratio=\frac{Rr}{Max\left(Rr\right)}. $$

*R*1, *R*2 and *R*3 represent the ratio of H3K4me1, H3K27ac and H3K4me3 signals of a given enhancer to the total signal of enhancers in the corresponding tissue, respectively. *Max(Rr)* represents the maximum value of *Rr* in a specific tissue/cell line.

### Identification of target genes and enhancers that possibly regulate the miRNAs

Previous studies have shown that the distance between enhancers and target genes mainly distributes within 100 kb (20). Therefore, a gene can be considered as enhancer target gene if the TSS of gene is located within 100 kb upstream and downstream from the center of enhancer. The potential enhancer–miRNA regulatory relationships were identified by the following formula described in the previous study (6):

$$ R=\left(G-M\right)/\left(G+M\right). $$

*M* is the distance between the center of enhancer and the closest miRNA. *G* is the distance between the center of enhancer and the closest gene. We regarded enhancer–miRNA pairs with 0 < R < 0.2 as candidate enhancer–miRNAs regulation.

### Identification of TF–enhancer, TF–miRNA and TF–gene regulations

We considered enhancer that overlaps with TFBS of a TF as candidate TF-regulated enhancer (TF–enhancer) in each tissue/cell line. The TSSs of gene were retrieved by processing GENCODE annotations v19, and the 5 kb upstream and 1 kb downstream from TSS were used as promoter region (31). If the TFBS has any overlap with the gene promoter, this gene will be considered to be a possible TF–gene. Moreover, we downloaded the data of miRNA TSS from FANTOM5 (28) and presume the 10 kb upstream and downstream of the TSS of miRNA serves as the promoter region of miRNA (31). The TF–miRNA could be identified if the miRNA promoter has more than one overlap with the TFBS.

### Identification of tissue-specific genes and miRNAs

The tissue-specific index (TSI) (32) was used to measure the tissue specificity of a gene or miRNA. In order to avoid the effect of the expression dysregulation between normal and matched tumor tissues/cell lines, the TSI values for normal and tumor tissues/cell line were calculated. The TSI was calculated by the following formula:

$$\tau =\frac{\sum_{i=1}^N1-{X}_i}{N-1}. $$

*N* is the total number of tissues and cell lines. ${X}_i$ is the normalized expression value of this gene/miRNA in different tissues or cell lines (normalization is performed through dividing the expression value of gene/miRNA in each tissue/cell line by the maximum expression value of this gene). The maximum value of τ is 1, and higher value of τ indicates higher tissue specificity of the gene/miRNA.

### Identification of the impact of SNPs on regulatory elements

The potential impact of SNPs within the enhancer or affecting TFBSs that located on an enhancer was explored if the SNP has any overlaps with corresponding TFBS or enhancer region. The eQTL data were retrieved from GTEx, and a high correlation between SNP and gene could be identified if the *q* value is lower than 0.05.

### System design and implementation

EnhancerDB was built mainly based on the following three components: nginx (https://nginx.org) web server, SQLite3 (http://www.sqlite.org/index.html) and Python-based backstage service. The web service was built on Flask v0.12.2 (http://flask.pocoo.org), a Python web application framework, and Peewee, a small, expressive Object Relational Mapper (ORM) that converts data between SQLite and Python. Moreover, our web interface was built on Bootstrap v4.0.0 (http://getbootstrap.com/), the most popular HTML, CSS and JavaScript framework. Datatables v1.10.16 (https://datatables.net) and echarts v3.0 (http://echarts.baidu.com/) were used to enhance the interactivity of web interface. We recommend using the latest versions of Firefox, Chrome or Safari web browser for the best experience.

**Figure 1:**
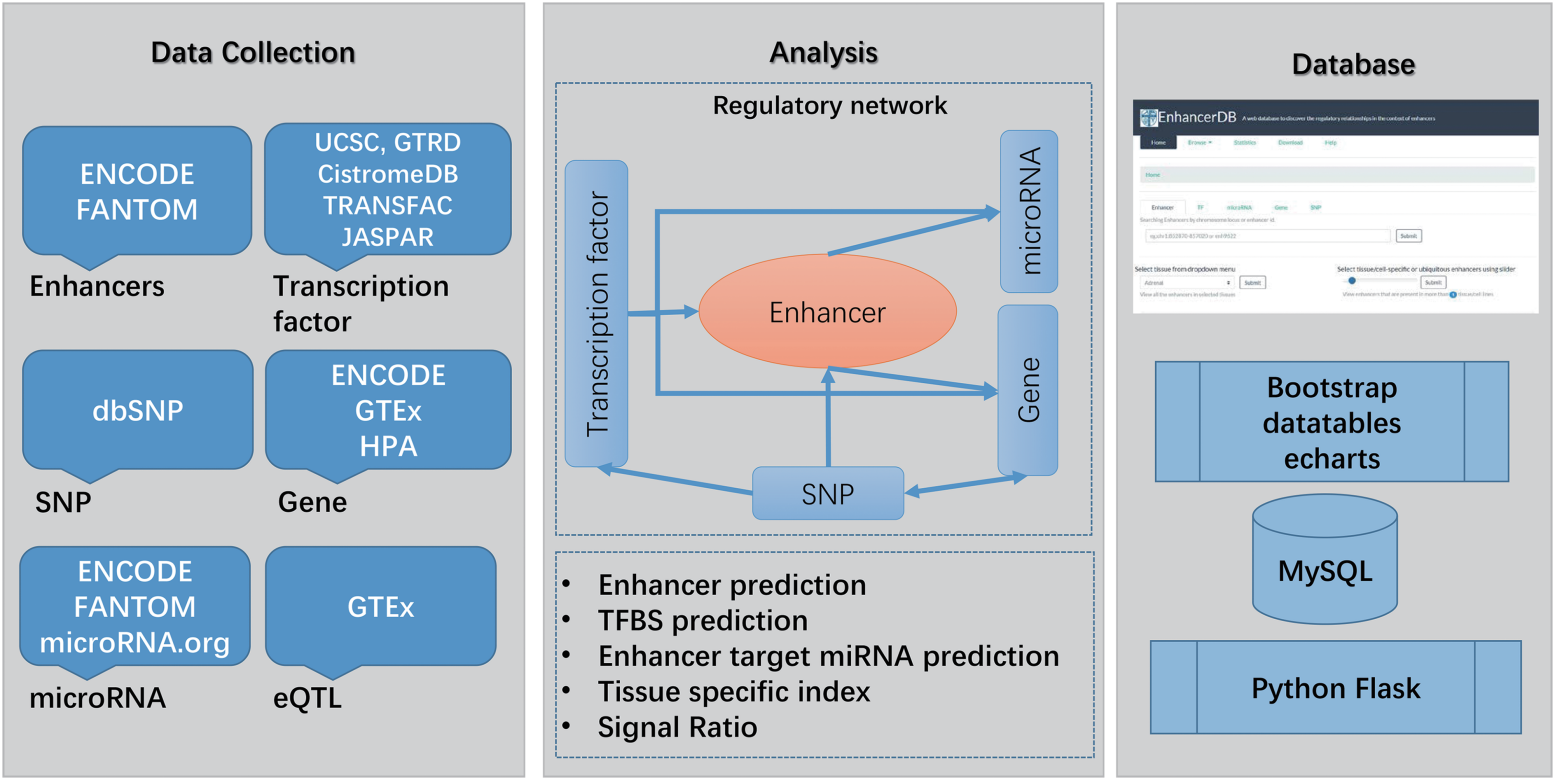
Main workflow of EnhancerDB. The data sources, workflow and database structure are displayed.

**Figure 2:**
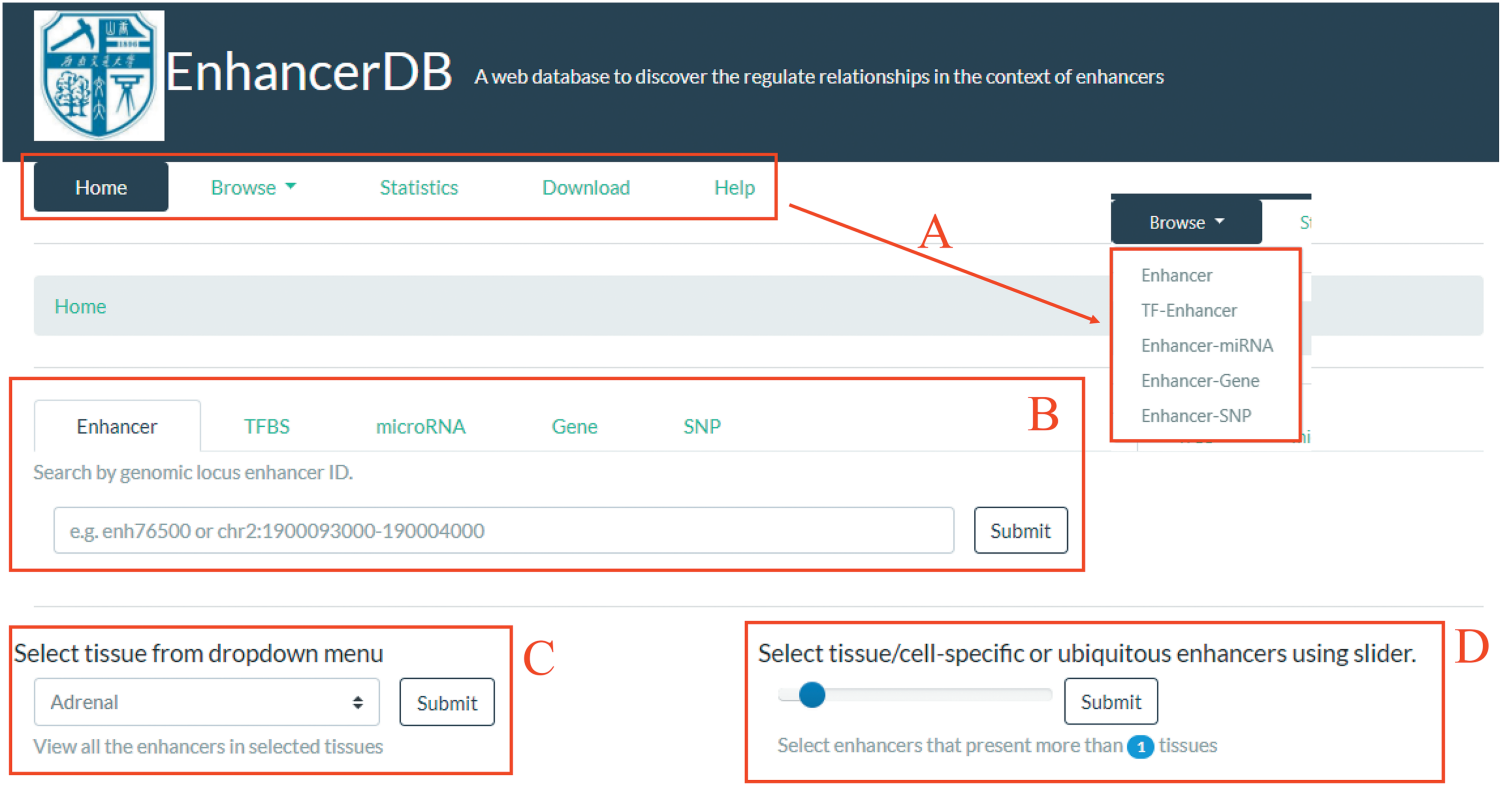
Home page of EnhancerDB. (A) Five main pages of EnhancerDB and the five entries under Browse subpage. (B) Search tabs of EnhancerDB. (C) Dropdown menu to explore enhancers in a tissue or cell line. (D) Slider to explore the tissue-specific or ubiquitous enhancers.

## Results and discussion

### Database content

In this study, we identified a total of 116 278 enhancers across 41 tissues/cell lines. Besides, EnhancerDB contains enhancers’ data in VISTA, a database consisting of experimentally validated enhancers in human and mouse, and FANTOM5, a project that defines enhancers by using cap analysis of gene expression through the determination of bidirectionally transcribed RNA. To distinguish enhancers from different sources, the prefix of enhancer ID were added for each enhancer name. In detail, IDs of enhancers identified by EnhancerDB, obtained from FANTOM5 and VISTA start with ‘enh’, ‘fantom’ and ‘vista’, respectively. We illustrated the distribution of enhancers identified in this study as well as those from VISTA and FANTOM with karyoploteR package (33). Supplementary data Figure S1 showed that our enhancers exhibited a high consistency with VISTA and FANTOM enhancers (Supplementary data Figure S1). Currently, our database contains 490 TFs, 1726 miRNAs, 23 334 genes and 11 381 519 SNPs in 41 normal and caner tissues/cell lines (Supplementary data Table S2). In total, 131 054 581 pairs of TF–enhancer, 17 059 pairs of enhancer–miRNA and 318 993 pairs of enhancer–gene regulation relationships were identified. Moreover, 4 039 558 pairs of TF–miRNAs regulation relationships and 1 059 695 pairs of TF–genes were found in 41 tissues/cell lines (Supplementary data Table S3). Finally, 119 938 pairs of eQTLs, involving 92 368 SNPs and 23 040 genes, were also explored. The main workflow of the database is illustrated in Figure 1.

**Figure 3:**
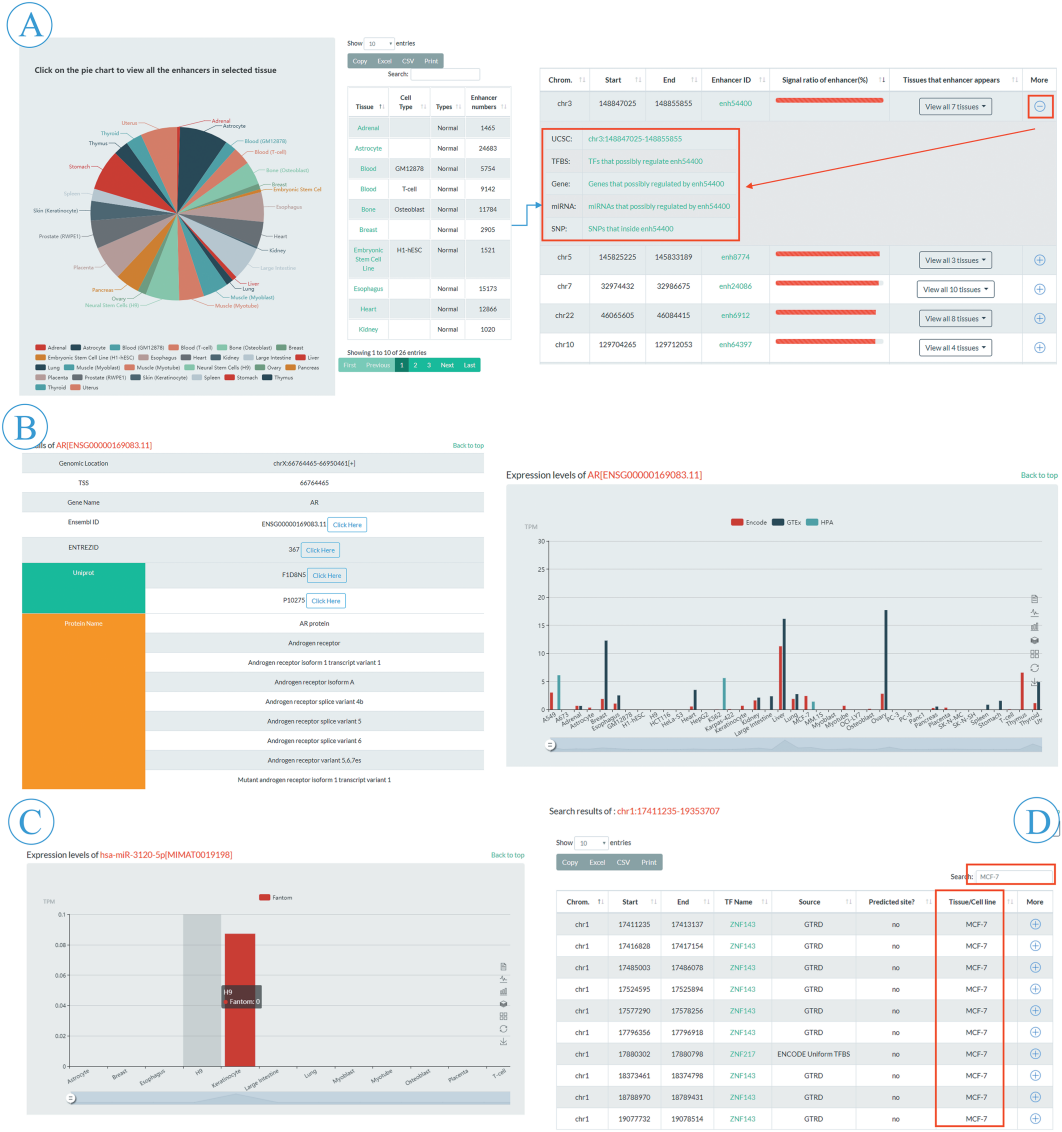
Browse and search flow of EnhancerDB. (A) Pie chart and information table of enhancers in 41 tissues/cell lines (left), including genomic loci, EnhancerDB id, Signal ratio of enhancers and tissues/cell lines name. ‘More’ column provides extra links to explore genomic features around enhancers, TF-enhancers, enhancer-genes, enhancer-miRNAs and SNPs within enhancers (right). (B). Detailed information of selected TF, including data source, expression level and external links, etc. (C) Expression level of miRNAs in multiple tissues/cell lines. (D) An example of secondary search for searching all TFBSs presenting in MCF-7 cell line in results using keyword “MCF-7”.

### Web interface

We developed a user-friendly web interface to help users to browse, search and download the regulatory relationships between enhancer and other regulatory elements. The web interface was split into the following five main pages: (i) browse, (ii) search, (iii) statistics, (iv) download and (v) help (Figure 2A). On the home page, we provided search tabs (Figure 2B) for users to explore enhancers, TFs, miRNAs, genes and SNPs of their interests. Moreover, there is a dropdown menu (Figure 2C) and a slider (Figure 2D) for users to view the enhancers in a selected tissue/cell line and to explore tissue-specific/ubiquitous enhancers, respectively.

### Browsing the database

The browse page was comprised of five entries, including enhancers, TF–enhancer, enhancer–miRNA, enhancer–gene and enhancer–SNP (Figure 2A). In the enhancer subpage, users can view all the enhancer information appeared in a specific tissue or cell line by clicking the hyperlinks on the pie chart (Figure 3A). A slider was also provided for users to set threshold of signal ratio to filter the enhancers with lower confidence. In the result table, besides the information of genomic loci and tissue/cell line, the ‘More’ column provides extra links to explore the genomic features around the enhancer, TF–enhancer, enhancer–miRNA and enhancer–gene, and SNPs within enhancer (Figure 3A). All the TFs in EnhancerDB were displayed on TF–enhancer web page and users can choose a specific TF to view detailed information, including genomic loci, TSS and external links to Ensembl, NCBI and Uniprot etc. There is also a ‘More’ column on the TFBS table for users to explore more information, including an external link to UCSC to view the genomic features around this TFBS, links to browse enhancers, miRNAs, genes regulated by this TF and SNP sites within this TFBS. At the bottom of the page, the histogram shows the expression level of TF in each tissue/cell line derived from ENCODE, GTEx and HPA (Figure 3B).

In the enhancer–miRNA or enhancer–gene subpage, users can browse miRNA or gene information such as genomic location, name, miRBase or Ensembl ID, TSI, enhancer–miRNA/gene, TF–miRNA/gene and expression level of the miRNA/gene (Figure 3C). Also, an external link to miRBase or Ensembl is provided for detailed information. Moreover, users can choose the miRNA/gene of interest by names or ID through the searching box at the top right of the table. Finally, users can obtain the genomic location, dbSNP ID, reference and alternative allele of different SNPs on the enhancer–SNP subpage. In the ‘More’ column, users can browse the SNPs within the enhancer or TFBSs, the gene associated with eQTL SNPs or click the link to explore other databases including dbSNP, RegulomeDB (34) and SNPedia.

### Searching the database

EnhancerDB provides ‘Search’ tab panels on the home page (Figure 2B). We offer various searching options for different elements, including genomic location of enhancer, EnhancerDB ID, miRBase ID, miRNA name and genomic location of miRNAs, as well as Ensembl ID, the gene name for genes. The genomic location and dbSNP ID are also supported when users perform an SNP search. Take the TFBS menu search using the location chr1:17411235-19353707 as an example, users can obtain information of all the TFBSs that overlap with chr1:17411235-19353707. Based on those searching results, if the user wants to further query TFBS which is only presented in MCF-7 cell line, they only need to input the keyword ‘MCF-7’ in the secondary search box (Figure 3D).

### Future development

In the next years, we will focus on collecting data for different species to construct a multispecies database. With the further amounts of data yielded by high-throughput techniques of multi-omics projects, we will continuously collect the latest data sets to keep our database up-to-date. In addition, the experimentally verified enhancers from the literatures will be added. We look forward to seeing our work promote further understanding of functions of enhancers.

## Author contributions

Zhiun Guo and Ran Kang designed and supervised the experiments. Ran Kang, Yiming Zhang, Junhua Meng and Yunjian Chang collected the data and designed and constructed this system. Qingqing Huang, Ruofan Ding and Lili Xiong were responsible for the data quality control. Ran Kang and Zhiyun Guo drafted the manuscript.

## Supplementary Material

Supplementary Data

## Notes

**Database URL**: http://lcbb.swjtu.edu.cn/EnhancerDB/
