# Supplementary material for: EnhancerDB: a resource of transcriptional regulation in the context of enhancers
Source: Database (Oxford). 2019 Jan 24;2019:bay141. doi: 10.1093/database/bay141 (PMC6344666; doi:10.1093/database/bay141)
Supplement: Supplementary Data [file bay141_supp.zip › Supplementary figure 1.docx]

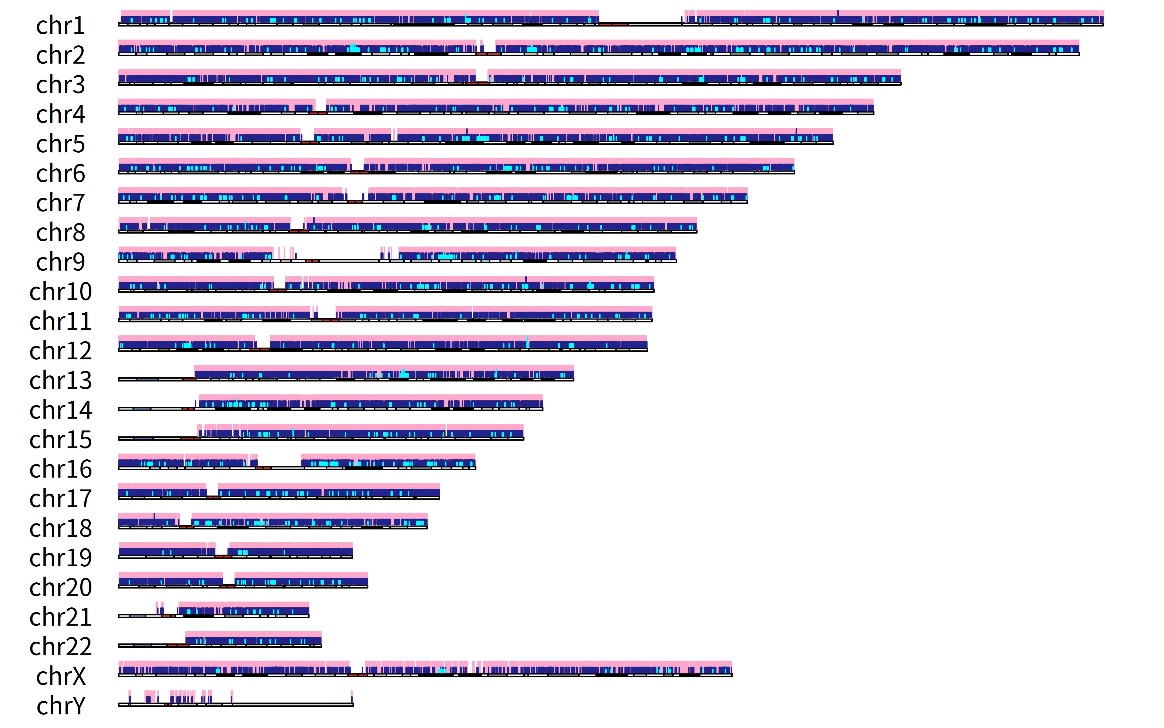


Figure S1. The genomic coverage of enhancers identified in this study and those obtained from VISTA and FANTOM5. Pink represents enhancers identified in this study. Blue represents the FANTOM enhancers. And cyan represents human enhancers in VISTA.
